# Macrophage polarization drives viral reservoirs in HIV-1 infection

**DOI:** 10.3389/fimmu.2026.1941085

**Published:** 2026-10-19

**Authors:** Aldo Emmanuel Calicio Glokpon, Sedaminou Judith Gbenoudon, Christopher Zaab-Yen Abana, Prince Adom Nartey, Jonathan Klutse, Helena Lamptey, Evelyn Yayra Bonney, George Boateng Kyei

**Affiliations:** 1 Laboratory of Immunology, Infectious and Allergic Diseases, Institute of Applied Biomedical Sciences, Faculty of Sciences and Technology, University of Abomey-Calavi, Cotonou, Benin; 2 Departments of Virology Noguchi Memorial Institute for Medical Research, College of Health Sciences, University of Ghana, Accra, Ghana; 3 Departments of Immunology Noguchi Memorial Institute for Medical Research, College of Health Sciences, University of Ghana, Accra, Ghana; 4 Medical and Scientific Research Centre, University of Ghana Medical Centre, Accra, Ghana; 5 Departments of Medicine and Molecular Microbiology, Washington University in St. Louis, St. Louis, MO, United States

**Keywords:** HIV reservoir, latency, macrophages, polarization, reactivation

## Abstract

Macrophages are cells of the myeloid lineage that play a very important role in innate immunity and tissue homeostasis. They have the capacity to respond to aggression from pathogenic agents and are the target of HIV-1 infection via their CD4 receptor and the CCR5 and CXCR4 co-receptors. In People With HIV (PWH), macrophages constitute a virological reservoir, making treatment with Antiretroviral Therapy (ART) difficult as they are resistant to the cytopathic effect induced by the virus, resist apoptosis and are in parts of the body with limited access, such as the brain. During HIV infection, macrophages are activated and polarized with the aim of eliminating the virus. Based on their polarization, they are classified into three main groups during HIV infection: M1 and M4 macrophages, which are classically activated, and M2 macrophages, which are activated alternatively. M2 macrophages are subdivided into several subtypes: M2a, M2b, M2c and M2d. M1 and M4 macrophages are pro-inflammatory and are stimulated by pro-inflammatory cytokines such as IL-2, IL-1r and TNF-α. M2 macrophages are anti-inflammatory and are stimulated by anti-inflammatory cytokines such as IL-4, IL-10 and IL-13. Macrophages constitute a very significant and long-lasting viral reservoir due to their intrinsic longevity. Furthermore, the accumulation of virions within Virus-Containing Compartments (VCCs) maintains the latent virus, thereby demonstrating their role as a critical, stable and long-lasting reservoir. However, the reservoir establishment and the persistence of the virus in polarized macrophages have not been fully explored. In this review, we examined macrophage polarization dynamics during HIV infection and explained how polarized macrophages contribute to the establishment of viral reservoirs. We also discuss how the acquisition of specific phenotypes may be a key factor in HIV reservoir formation. Thus, providing important information to guide reactivation of HIV from macrophages, a critical step in the Shock-and-Kill HIV cure strategy.

## 1 Introduction

HIV remains a Public Health problem despite the efforts of the World Health Organization (WHO) through United Nations Program on HIV/AIDS (UNAIDS) and other international organizations. Combination antiretroviral therapy (cART) has improved disease prognosis of patients and slowed viral transmission by reducing the viral load to undetectable level (1). Although cART has shown high efficacy, it has faced some challenges, including persistence of the virus in reservoir cells and restricted access to certain organs such as the brain. Thus, when ART is stopped, the virus rebounds, making it difficult to completely eliminate the virus from the body (2, 3). The establishment of HIV-1 latent reservoir in humans occurs rapidly and this proviral reservoir is stable and persists during cART treatment (4). The main targets of HIV infection are CD4 T cells thus decreasing their numbers, due to cell death, weakens the immune system of people with HIV (PWH). The persistence of HIV is driven by its ability to infect diverse cells, including memory CD4^+^ T cells (TCM, TTM, and TEM), dendritic cell subsets (FDCs, mDCs, and pDCs), astrocytes, microglia, hematopoietic progenitor cells, and renal tubular epithelial cells, contributing to viral persistence despite antiretroviral therapy (5–8). Additionally, other myeloid cells like monocytes and macrophages can be infected by HIV (9). The ontogeny of tissue-resident macrophages allows them to be classified into two categories: those derived from the embryonic yolk sac or foetal liver, which have a long lifespan and are self-renewing, as opposed to macrophages arising from the differentiation of bone marrow-derived monocytes, which have a short lifespan (10, 11). Macrophages are key players in human immunity due to their ability to live in almost all tissues, macrophages play a crucial role in the establishment and persistence of the HIV-1 reservoir (12). Their susceptibility to infection depends on their permissiveness to resistance, tissue localization and polarization profile (13). In the brain, microglia constitute the main myeloid reservoir for HIV-1, maintaining a state of latency with low proviral reactivation, which promotes viral persistence despite antiretroviral therapy (14, 15). Known as Kupffer cells in the liver, they are susceptible to HIV-1 infection and harbor persistent proviral DNA in individuals receiving ART, with impaired or limited ability to replicate (16). As for alveolar macrophages, which are capable of harboring HIV-1 DNA or RNA whilst on effective antiretroviral therapy, their abundance and cell-to-cell contact within the alveolar space contribute to the local persistence of the virus and its low level of replication in the pulmonary compartment (17, 18). Whilst macrophages in the intestinal lamina propria are resistant to productive HIV-1 infection, those in the vaginal mucosa remain highly susceptible to it, and macrophages in the male genital mucosa constitute the major reservoir of competent viruses that can be reactivated during ART (19, 20). This polarization confers a specific phenotype on these cells and enables them to perform their functions. Following their infiltration into tissues, macrophages can polarize in response to stimuli that may be internal factors such as cytokines or external factors derived from bacteria such as lipopolysaccharide (LPS). Cytokines such as IFN-γ and TNF-α direct macrophages toward the pro-inflammatory M1 phenotype, whereas anti-inflammatory M2 macrophages are induced by exposure to IL-4, IL-13, IL-10 or glucocorticoids (21). M1 macrophages are classically activated and produce pro-inflammatory cytokines such as TNF-α and IL-12, thereby helping the immune system to eliminate pathogens, whereas alternatively activated macrophages M2, play anti-inflammatory roles and contribute to wound healing (22). Furthermore, during HIV-1 infection of macrophages, there is an increased production of chemokines associated with the M1 phenotype, notably CCL3, CCL4 and CCL5, accompanied by a reduction in M2-associated markers such as CD163, the mannose receptor CD206, CCL18 and IL-10 (23). During HIV infection, the dynamics of macrophage polarization play an important role in viral elimination. Viral latency in macrophages; a reversible non-productive state occurring in the weeks following infection, has been demonstrated *in vitro* through the reactivation of viral production in infected macrophages after Toll-like receptor 4 (TLR4) stimulation by LPS (20). However, not much has been done beyond that. In this review, we explore in-depth how HIV influences macrophage polarization and function as viral reservoirs. A clearer understanding of the interaction between macrophage polarization and HIV persistence within macrophages will help inform strategies aimed at HIV cure/remission.

## 2 Macrophages: a key player in the HIV reservoir

Macrophages intervene as first line against foreign bodies that are pathogenic and invasive. Beyond this defensive role, their regenerative capacity, originating from embryonic progenitor cells and circulating blood monocytes, equips them to sustain homeostasis; a function that underlines their distribution across all body compartments, and are able to respond rapidly to environmental changes and infections (24–26). It has been demonstrated that HIV continues to exist in cellular reservoirs due to poor penetration of antiretrovirals (ARVs) into viral sanctuaries in target organs, and establishment of viral latency or cryptic viral replication (9). While CD4^+^ T cells were initially identified as the primary viral reservoir, evidence now implicates myeloid-lineage cells, particularly macrophages, as equally significant. A study carried out on CD64+ and CD68+ intestinal macrophages from people with HIV on highly active antiretroviral therapy reveals the presence of detectable levels of p24 and HIV DNA in macrophages (27). Macrophages and microglia, as HIV reservoirs, use a novel mechanism to prevent apoptosis at a very early stage (28). In microglial cells, two forms of latency have been described to explain the persistence of HIV-1 in the cells. Pre-integration latency, which occurs prior to integration of the HIV-1 genome into the host genome, and post-integration latency lead to persistence of infection (9, 15). Macrophages are versatile innate immune cells that by homeostatic maintenance and tissue adaptation, are present in all body compartments (29, 30). The detection of viral products within macrophages despite ART, combined with their capacity to evade apoptosis and establish latency, positions them as a critical obstacle to cure, an obstacle whose tissue-specific dimensions are examined in the following section.

### 2.1 Localization of infected macrophages in different tissues and organs like reservoir

Infected macrophages have been detected across a broad range of tissue compartments, including the central nervous system (CNS), gastrointestinal tract, lymph nodes, liver, and reproductive mucosa. Each represents a pharmacologically distinct reservoir niche with implications for viral persistence. In the CNS, HIV-1-infected macrophages are responsible for HIV-associated dementia (HAD) in advanced stages of the disease (9, 15, 31–34). DNAscope analysis of 29 post-mortem brains shows that HIV-1 DNA persists in the frontal white matter (FWM), the basal ganglia (BG) and the corpus callosum (CC) of patients on suppressive antiretroviral therapy, with or without HIV-associated neurocognitive disorders (35). The isolation of Brain myeloid cells (BrMCs) from post-mortem samples of PWH on ART demonstrates that microglia constitute a persistent reservoir of HIV-1, harbouring replication-competent viruses with macrophage tropism (36).The gastrointestinal tract (GIT) is a primary site of HIV infection, replication and dissemination due to the latent state of the virus. Myeloid lineage cells from gut-associated lymphoid tissue (GALT) of people with HIV on ART have been found to harbor HIV (12, 27). *In situ* hybridization and immunohistochemical analyses carried out on Rhesus macaques revealed that macrophages present in lymph nodes, the spleen, the gastrointestinal tract, the liver and the kidneys produced more than 95% of the virus, thus maintaining a high plasma viral load (37). In 2010, Zalar et al., detected HIV DNA in the macrophages of 9 out of 20 patients in duodenal tissue from HIV intestinal biopsies (38). In addition, HIV is able to replicate in urethral macrophages in people with suppressed viremia tested by DNA measurement (39). Other studies have reported HIV infection in the liver through the detection of viral RNA, DNA and glycoproteins in Kupffer cells of PWH and furthermore, vertical transmission of HIV-1 from mother to child during pregnancy is effective through placental Hofbauer macrophages (40). HIV has been detected in macrophages from the lymph nodes (41) of infected humans and they are also present in semen, urethra, prepuce, vaginal mucosa, rectal mucosa and cervical mucosa, making them a potential source of HIV transmission during sexual (42). The localization of the virus in macrophages that have differentiated in several organs makes its elimination very complicated. In these different organs, ARVs have limited access to their targets, especially in the brain, and therefore cannot exert their optimal pharmacological effect. The breadth of this tissue distribution, combined with variable ARV penetration across compartments, creates conditions that favor viral persistence. A persistence that is further sustained by the exceptional longevity of macrophages relative to other infected cell types.

### 2.2 Macrophage longevity, contributing to the persistence of the viral reservoir

Under physiological conditions, uninfected memory and naïve T cells exhibit significant kinetic heterogeneity. Naïve T cells have a lifespan of several months or even years, whilst that of memory T cells ranges from a half-life of 30 to 160 days to a long lifespan (8–15 years) (43, 44). At steady state, peripheral T lymphocytes exhibit prolonged survival exceeding 14 days, whilst macrophages of circulatory origin display a condition-dependent lifespan, characterized by a half-life of approximately 14 days in normal tissues (45).Unlike activated lymphocytes infected with HIV-1, which have a relatively short lifespan of around 0.8 to 1.1 days, productively infected macrophages retain their viability and viral productivity for at least 30 days (31). The myeloid reservoir of HIV may be clinically relevant as it has a potentially long lifespan, is relatively resistant to the cytopathic effects of HIV infection and is resistant to destruction by CTLs (39). Macrophages contain large amounts of non-integrated HIV DNA that persists for longer periods (2 months) and could potentially contribute to viral persistence (46). When infected, microglial cells are much more resistant to cytopathic effects and apoptosis (15). Contributing to this persistence at the subcellular level, multivesicular bodies (MVBs) provide a non-degradable compartment in which HIV-1 may remain infectious for up to seven weeks after infection (47). To survive in macrophages, the virus decreases the expression of pro-apoptotic proteins while increasing the expression of anti-apoptotic regulatory proteins, such as those of the BCL2 family and X-linked inhibitors of apoptosis (XIAP) (48, 49). For example, in other diseases like acute coronary syndrome (ACS), the chronic infection induces a senescence of premature CD4+ T cells, which may be responsible for the development and the progression of the disease of ACS (50). The control of internal restrictive factors in macrophages by the presence of the virus, preventing their self-destruction under specific conditions, favors the persistence of the reservoir. These recent findings confirm that cellular longevity is not a fixed property, but is dynamically adjusted by the specific infectious or inflammatory context, thereby providing a useful comparative basis from which HIV-induced alterations in the half-life of lymphocytes and macrophages can be interpreted. Collectively, the upregulation of anti-apoptotic proteins, resistance to cytopathic effects, and subcellular compartmentalization suggest that infected macrophages have acquired, or have been co-opted into a survival program, that directly serves viral persistence. Understanding how HIV establishes and sustains this durable intracellular state requires closer examination of the mechanisms governing viral entry, replication, and latency within macrophages. These mechanisms are discussed in the following section.

## 3 Macrophage polarization

### 3.1 Types of macrophage polarization

Tissues macrophages are highly heterogeneous and their differentiation is determined by tissue-specific combinations of individual cytokines, transcription, and growth factors. As shown in **Table 1**, macrophages adopt distinct activation states depending on the stimuli in their microenvironment, ranging from pro-inflammatory (M1, M4, Mox) to anti-inflammatory. Macrophage polarization is driven by cytokine signals in the microenvironment that direct cells toward functionally distinct phenotypes, broadly classified as pro-inflammatory M1 and anti-inflammatory M2. The presence of IFN-γ and TNF-α, for example, can switch macrophages to an M1 phenotype while IL-4 or IL-13 will switch it to M2a and IL-10 or glucocorticoids will switch it to M2c (21, 24, 29, 67). In addition, M2 macrophages are classified into subtypes with distinct phenotypes: M2a, M2b, M2c and M2d, in response to specific stimuli (51, 68). The M2b phenotype, a subtype of M2 macrophages also known as regulatory macrophages, promotes tumour development and parasitic, bacterial and fungal infections in cancer and other infectious diseases (59). By combined exposure to immune complexes and TLR agonists or IL-1R agonists, M2b macrophages express high levels of CCL1 and TNF superfamily member 14 (TNFSF14) and pro-inflammatory cytokines as IL-1β, IL-6 and TNF-α (60, 69). The T helper 1 (Th1) cell cytokines such as IFN-γ, along with stimuli including LPS and GM-CSF, induce the M1 phenotype (25), whereas the M2 phenotype is induced in response to T helper type 2 (Th2) cytokines, such as IL-4 and IL-13, as well as anti-inflammatory cytokines including IL-10 and TGF-β and factors such as M-CSF (70, 71). M1 macrophages exhibit strong antigen-presenting capacity and can drive Th1 immune responses, whereas, M2 macrophages, are characterized by high expression of CD10, CD163, CD206, C-type lectin domain family 4 member L (CD209), and arginase 1 (Arg1) and by the release of large amounts of anti-inflammatory cytokines such as IL-10, TGF-β (72). Other specialized macrophage subtypes, such as M4, Mhem and Mox, have been described in atherosclerosis and may represent an activation axis distinct from the M1/M2 model (62). Recent studies have shown that M4-type macrophages constitute the major tissue reservoir of replication-competent HIV-1 in the genital mucosa of ART-suppressed individuals, with the M4-derived alarmin S100A8 driving glycolysis-dependent reactivation of viral production from these cells (20).

**Table 1:** Macrophage polarization as a function of stimuli and inflammatory role.

Types of macrophages	Stimuli	Inflammatory role and activation mode	References
M1	INFγ, TNF-α, GM-CSF, LPS	Pro-inflammatory Classique activation	(51–54)
M2a	IL-4, IL-13, M-CSF	Anti-inflammatory Alternative activation	(51, 53, 55–58)
M2b	TLR stimulation, IL-1ra	Anti-inflammatory Alternative activation	(51, 55, 59, 60)
M2c	IL-10, TGF-β1 glucocorticoids	Anti-inflammatory Alternative activation	(56, 57, 61)
M2d	IL-10, TGF-β, VEGF	Anti-inflammatory Alternative activation	(56, 57, 61)
M4	CXCL4, TNFα, M-CSF	Pro-inflammatory Classique activation	(20, 62, 63)
Mhem	IL-10	Anti-inflammatory **-–**	(64–66)
**Mox**	**QxPAPC**	**Pro-inflammatory** **-–**	(63, 64, 66)

Mox phenotype, is induced by oxidised phospholipids (QxPAPC) rather than by cytokines and Mhem macrophages are induced by haem.

### 3.2 Activation pathways involved in macrophage polarization

Macrophage polarization toward M1 or M2 phenotypes is orchestrated by a network of intracellular signaling cascades; including Janus Kinase/Signal Transducer and Activator of Transcription (JAK/STAT), Nuclear Factor-κB (NF-κB), and Mechanistic Target of Rapamycin (mTOR) pathways, each responding to distinct stimuli. This acquisition of a new functional phenotype (polarization) results in a heterogeneous macrophage population. Indeed, following activation of membrane receptors by cytokines or LPS, the pathways that drive into M1 phenotype mainly involves l’axe JAK/STAT1, NF-κB, IRF5. To differentiate and carry out their functions, macrophages undergo metabolic reprogramming at the same time. For example, in the case of the JNK/COX-2/HIF-1α axis, HIV-1 induces both M1 polarization and an increase in glycolysis in macrophages, because inhibition of glycolysis blocks this polarization, indicating that the increase in glycolysis promotes M1 polarization (73). The JAK/STAT3 signaling has several functions such as proliferation of CD4+ T cells, polarization of Th1 cells, activation of cytotoxic T cells, generation of natural killer cells and differentiation of macrophages and dendritic cells (74). Certain pro-inflammatory cytokines produced during the Th1 response, such as IFN-γ and TNF-α, induce the polarization of macrophages into the M1 phenotype, showing that JAK/STAT3 signaling is involved in M1 polarization. In the brain, microglial homeostasis is maintained by autocrine TGF-β and the IL-34/Colony-Stimulating Factor 1 Receptor (CSF1R) pathway, and inflammatory activation by IFN-γ, TNF-α and neuronal Damage-Associated Molecular Patterns (DAMPs) overrides this regulatory pathway to induce an M1-like state (75, 76). Furthermore, in the lung, alveolar macrophages require Granulocyte-Macrophage Colony-Stimulating Factor (GM-CSF) from type 2 alveolar epithelial cells, as well as TGF-β, which in turn leads to the induction of Peroxisome Proliferator-Activated Receptor gamma (PPAR-γ), which subsequently drives alveolar macrophages towards a pro-inflammatory state (76, 77), resulting in the M1 phenotype. The Programmed Cell Death Protein 1 (PD-1) deficiency in mouse macrophages induces M1 rather than M2 polarization by increasing phosphorylated-STAT1/p-NF-κB p65 expression and downregulating p-STAT6 (78). Stimulation of NF-κB signaling polarizes macrophages into M1 and its inhibition promotes macrophage polarization into M2 (79). Estrogen receptor-α (ERα) knockdown promotes NF-κB/p65 transcriptional activity and induces M1 polarization in women (80). Finally, activation of Interferon Regulatory Factor 5 (IRF5) expression polarizes macrophages towards an M1 phenotype (81). For the polarization of macrophages into M1, the STAT1-NF-κB pathway is activated and the latter goes into the nucleus at the promoter sites of the genes governing the M1 phenotype. Concern the polarization into M2, the principal axis involved is STAT6, Peroxisome Proliferator-Activated Receptor-γ (PPAR-γ), mTOR. When the Inositol-Requiring Enzyme 1 alpha/X-box Binding Protein 1 (IRE-1a/XBP-1) signaling pathway is inhibited, M1 polarization is blocked and Protein kinase RNA-like Endoplasmic Reticulum Kinase (PERK) knockdown facilitates the polarization of macrophages into M2 via STAT1 and STAT6. Deficiency of lysosomal adaptor protein (Lamtor1), for example, causes amino acid deprivation or inhibition of v-ATPase and the mTOR complex, which is essential for M2 polarization and thus will direct the effect of polarization in M1 to a greater extent (82). Upregulation of Serine/Arginine-Rich Splicing Factor 10 (SRSF10) induced by lactate accumulation following histone lactylation promotes the polarization of M2-type macrophages (83). IFN- γ activates the JAK/STAT1 signaling cascade which induces the release of pro-inflammatory mediators by macrophages and when STAT1 activity is inhibited, it can lead to M2 polarization (52). When Matrix metalloproteinase-9 (MMP-9) is activated, polarization is directed towards the M1 phenotype and when inhibited, it favours the M2 phenotype (79). *In vitro* experiments demonstrated that biocompatible ionized air (BIA) modulates the polarization of M1 macrophages toward the M2 phenotype via the ROS-mediated STAT6 pathway (84). NF-κB, MAPK, PI3K/Akt and IRF are molecular complexes involved in M2b macrophage polarization (59). The STAT6, PPAR-γ, mTOR pathway is essential for the polarization of macrophages into the M2 phenotype; therefore, to regulate this pathway, we will need to target one of its components. Regarding M4 macrophages, their differentiation is induced by the signalling pathway regulated by CXCL4 (platelet-derived growth factor 4). Indeed, the chemokine CXCL4 binds to glycosaminoglycan-type proteoglycans or chondroitin sulfate on the surface of monocytes rather than to a conventional G-protein-coupled receptor and subsequently triggers a transcriptional program characterized by the co-expression of CD68, S100A8, and MMP7, which are markers distinguishing M4 cells, accompanied by an irreversible and complete loss of the scavenger receptor CD163 (64, 85). The loss of CD163 abolishes hemoglobin clearance and prevents the induction of the atheroprotective enzyme hem oxygenase-1, a functional signature that mechanically distinguishes M4 which is present in male genital mucosa cells from the M1 and M2 lineages (20, 64). Furthermore, the synergy between the TLR4-dependent TBK1–JNK–AP-1 pathway and the NF-κB factor enables CXCL4 to drive the pro-inflammatory and fibrotic gene program specific to M4 macrophages. This confirms the pro-inflammatory nature of M4 macrophages and their ability to be induced by the TLR4/LPS signal (86). After polarization into M1 or M2, the regulation of these macrophage subtypes must maintain a balance to prevent any dysregulation of the immune response. The Pathways that regulate the balance between these two types phenotypes are l’axe TLR2/STATs, PERK/eIF2a. Alteration of the STATs signaling pathway via TLR2 inhibits the differentiation of monocytes into M1 or M2 macrophages showing its importance in macrophage polarization regulation (22). In contrast, the gut harbors tolerogenic M2-like macrophages, which are maintained by TGF-β and IL-10 under normal conditions, and pro-inflammatory M1 macrophages, which emerge following microbial stimulation when this TGF-β/IL-10 signaling is lost (87). In the presence of IFN-γ, STAT-1 activity is important for macrophage polarization towards the M1 profile and, on the other hand, STAT-6 and PPAR-γ activity in the presence of IL-4/IL-13 regulates several genes such as Arg1, CD206 and Ym1 that are associated with macrophage polarization towards the M2 profile (67). The PERK-eIF2a-ATF4 pathway is then involved in the regulation of macrophage polarization (72). Therefore, the convergence of these signaling pathways on a small number of transcriptional regulators, especially STAT1, STAT6, and NF-κB, suggests that selective modulation of macrophage polarization may be achievable through targeted pathway intervention, with direct implications for reservoir management in HIV infection.

## 4 Mechanisms of HIV infection and persistence in macrophages

### 4.1 Routes of entry of HIV into macrophages

Macrophages are susceptible to HIV-1 infection through both classical receptor-mediated fusion and a range of alternative cell-to-cell transmission mechanisms that bypass conventional entry barriers. To bind to the host cell, the virus uses its glycoprotein gp120 to bind to the CD4 receptor and then its glycoprotein gp41 to bind to the CCR5/CXCR4 co-receptor to fuse with the host cell membrane (46, 47). The expression of these receptors varies from one tissue to another. Vaginal macrophage populations expressed CD4 and CCR5/CXCR4 similarly to blood monocytes, but intestinal macrophages expressed little or no detectable CD4 and CCR5/CXCR4 (27, 39). Beyond receptor-mediated fusion, macrophages can be infected through intercellular mechanisms, including tunneling nanotubes (TNT) formation. HIV-1 enters macrophages via an endocytic pathway distinct from conventional macropinocytosis, as it depends on Na(+)/H(+) exchange, actin rearrangement, dynamin, Rho GTPases and Pak1, but is independent of PI3-kinase and myosin II (88). Macrophages can become infected via cell-to-cell transmission through capture and selectively engulf HIV-1-infected CD4+ T cells, leading to effective infection of the macrophages (89). Fusion with acutely infected CD4+ T cells, produces viable heterokaryons, and phagocytosis of viral particles released by T lymphocytes enables infection at high multiplicity (47). Additionally, down-regulation of CD4 receptors induced by phosphatidylcholine liposomes in primary human macrophages reduces HIV-1 entry, thereby reducing the viral reservoir (90). The p53 protein plays a very important role in enhancing the antiviral activity of macrophages by down-modulating CCR5 expression and HIV-1 entry mediated by miR-103/107 (13). The multiplicity of these entry mechanisms, from direct receptor binding to cell-to-cell transfer, means that blocking any single pathway is insufficient to prevent macrophage infection, underscoring the challenge of reservoir clearance.

### 4.2 Effects of HIV infection on macrophage polarization/phenotype

Given their prevalence in various infectious contexts, M1 and M2 macrophages are particularly important in the context of HIV infection, where their polarization directly influences the formation of res`ßervoirs. As illustrated in **Figure 1**, unpolarised (M0) macrophages can be driven by pro-inflammatory stimuli such as IFN-γ, TNF-α, GM-CSF and LPS towards the classically activated M1 phenotype, which is characterised by increased latency but reduced reactivation of HIV-1, or by CXCL4, TNF-α and M-CSF towards the pro-inflammatory M4 phenotype, whereas IL-4, IL-13 and M-CSF promote alternative activation towards the M2 phenotype and its subtypes (M2a-d), and both M2 and M4 cells display increased latency together with a greater capacity for reactivation, so that the polarization state of macrophages determines the size and inducibility of the viral reservoir. This polarization imbalance is further sustained by immunometabolic reprogramming, whereby HIV-1 exploits macrophage metabolic states to reinforce its reservoir (91). HIV-1 induces a polarization of macrophages towards a pro-inflammatory M1 phenotype, thus contributing to the state of chronic activation of the immune system which is a major factor in the progression of HIV-1 disease (23, 63, 92). HIV infection of macrophages depends, among other things, on the state, level of maturation and type of activation of the cytokines present in their microenvironment. In HIV-1 infection, polarization of macrophages *in vitro* into M1 cells prevents infection and polarization into M2 inhibits viral replication at a post-integration level, then the M2a subtype with a high level of DC-like lectin expression facilitates virus transmission to the CD4 T cell, and in the M2c subtype, inhibition occurs after reverse transcription of HIV-1 (62, 63, 93). M1 polarization of *in-vitro* differentiated primary MDM determines a transient, but profound restriction of HIV-1 replication affecting multiple (entry and post entry) steps in the virus life cycle likely involving the upregulated expression of APOBEC3A (94).Whilst M1 polarization exerts a powerful inhibitory effect on HIV-1 infection by reducing CD4 receptor expression, saturating co-receptors through the secretion of chemokines (CCL3/4/5) and reducing the viral DNA load by more than 90%, M2a polarization, on the other hand, does not impede either the integration of the viral genome or the expression of viral proteins (95). Certain pro-inflammatory cytokines such as IL-2 inhibit HIV-1 replication in macrophages by down-regulating CD4 and CCR5 expression (96, 97). Polarized macrophages in M1 have been shown in one study to promote a very high level of restriction of HIV-1 replication, with a very low level of virus production close to undetectable levels (98). A study has shown that M4 macrophages, through S100A8 and glycolysis, are the main viral reservoirs in mucous tissue (20). In the pulmonary alveoli, anti-inflammatory CD163^+^-labelled alveolar macrophages were reduced and pro-inflammatory CD163-CCR7^+^-labelled macrophages were increased, with an increase in inflammatory cytokines and increased macrophage activation (92). Latently HIV-infected MDMs (MLat 1D5) show overall reduced surface expression of CD11b (M0), CD80 (M1), CD209 (M2), CD4, HLA-ABC and HLA-DR compared with uninfected MDMs/THP-1 (99). In addition, brain myeloid cells harboring the virus predominantly displayed microglial markers, in which up to 99.9% of the BrMCs were TMEM119+ microglia (36). However, the M1and M2 classification is operational rather than a fixed state, and this represents a limitation of the strict classification of macrophages. Cassetta et al. demonstrated that the upregulation of the M1 marker (CD80/CD86) in HIV-1-infected MDMs is transient and stimulus-dependent (94), whilst Jiménez et al. found that *In vitro* polarized MDMs with IFN-α and IFN-β express CD163 which is M2 marker (21). Thus, macrophage polarization after latency shows that pro-inflammatory M1-type macrophages suppress HIV reactivation, whereas M2-type macrophages promote it (99). Of all these interactions, very few M1s form a reservoir due to their strong restriction of the virus, unlike M2. The provirus in M1s will also be more difficult to reactivate by latency-reversing agents (LRAs) than in M2. Together, these polarization-dependent differences in viral restriction and reactivation potential suggest that macrophage phenotype must be accounted for in any reservoir-reduction strategy, a challenge that implicates the signaling pathways governing polarization itself.

**Figure 1:**
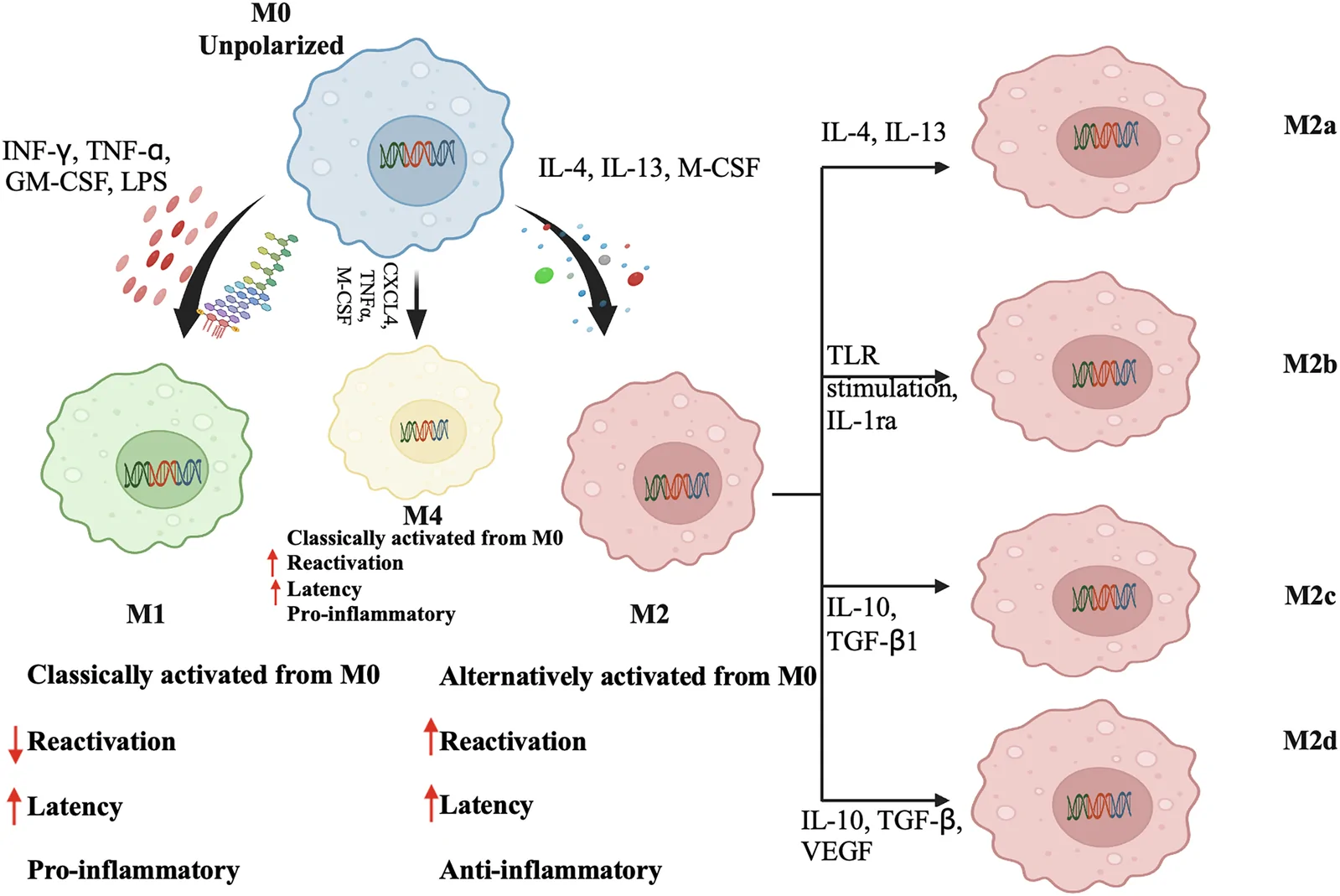
Macrophages polarization and their impact on HIV-1 infection: Whether the macrophages M1, M2 and its subtypes or M4 constitute a diverse reservoir in the body and the reactivation capacity of the latent provirus also depends on the phenotype. Thus, macrophages acquire a pro-inflammatory character when stimulated by pro-inflammatory cytokines such as: INF-γ, TNF-α, GM-CSF, or anti-inflammatory ones of this nature such as: IL-4, IL-13, M-CSF, IL-10, TGF-β1. https://BioRender.com/1x89k5p.

### 4.3 Particularities of viral replication in macrophages

Viral replication in macrophages is shaped by cell cycle state, polarization phenotype, and macrophage-specific molecular co-factors: features that collectively distinguish macrophage infection from replication in CD4+ T cells. At the cell cycle level, the transition from the G0 to G1 phase constitutes a gateway for HIV-1 entry because macrophages in the G1 state inactivate the antiviral restriction factor SAMHD1 by phosphorylation (24). Following entry, HIV-1 can establish three mechanistically distinct outcomes in macrophages that should not be used interchangeably. HIV-1 establishes a latency state in MDMs with integrated but transcriptionally silent provirus due to reduced NF-κB, RNA polymerase II and Positive Transcription Elongation Factor b (p-TEFb) recruitment plus non-permissive chromatin, and this transition depends on type I IFN signaling (100). A human monocyte-derived macrophage quantitative viral outgrowth assay (MDM-QVOA) and myeloid IPDA revealed that 40% of people on long-term ART carry reactivatable latent HIV in monocytes/MDMs that can persist for years and produce infectious virus (46). In addition, macrophage polarization is thought to have an impact on HIV infection. M1 showed more robust inhibition of virus replication, while M2 reduced HIV-1 production and M2 macrophages were able to live longer than M1 (101). In the absence of CD4^+^ T cells, long-lived HIV-infected perivascular macrophages were shown to contain replication-competent HIV proviral DNA, supporting persistent and permissive HIV infection (48). MDM transition to viral latency is mediated by type I IFN signaling, as inhibiting type I IFN signaling or blocking type 1 IFN prevents the establishment of latent infection (100). During *in vitro* studies, IFNγ and TNFα stimulated M1 MDMs inhibited HIV-1 viral DNA synthesis, proviral integration, and transcription in comparison to unpolarized MDMs through an upregulation of APOBEC 3A in M1 MDMs (102). Once the virus genome has entered the host, it must be replicated. In macrophages, one of the molecules that promotes this replication is cyclin L2, which interacts with kinase 1A (DYRK1A), which has dual specificity regulated by tyrosine phosphorylation (103). In short, the resistance of macrophages to the cytopathic effects of the virus after replication, unlike T lymphocytes, enables them to act as a long-term viral reservoir. A further distinguishing feature of macrophage replication is the assembly of virions within internal virus-containing compartments (VCCs), which shelter nascent particles from immune recognition and ARV access, hence makes them a very formidable and important reservoir in persistence.

### 4.4 Role of cellular and viral factors in the establishment and maintenance of viral latency in macrophages

The establishment of HIV latency in macrophages reflects a dynamic contest between host restriction factors; including SAMHD1, APOBEC3G, and Tetherin, and viral accessory proteins like Vpu, Vif, Nef and Vpr that systematically neutralize their antiviral activity. Following dephosphorylation, the SAMHD1 complex becomes a macrophage restriction factor, preventing active replication (104) of the virus by reducing the level of dNTP required for viral replication (24). Pre-integration latency is strongly regulated by host restriction factors such as SAMHD1, APOBEC3, MX2 and PAF1, and post-integration latency occurs after integration of HIV proviral DNA into the host genome, followed by inhibition of HIV gene expression (9, 46). The restriction factor APOBEC3G (A3G) is a cytidine deaminase that is targeted by the virion-associated accessory protein, Vif, which degrades it (101, 105) and Histone Deacetylase Inhibitors (HDACi) are inhibitors that activate the antiviral activity of SAMHD1 through cell cycle regulation in macrophages. In addition, HIV-1 has less cytopathic effect on macrophages than on CD4 T cells due to the non-activation of caspases, effectors of apoptosis, or may reduce the regulation of the death ligand TRAIL (TNF-related apoptosis inducing ligand) (24). The viral protein Vpr induces translocation from the cytoplasm to the cell nucleus and leads to cell cycle arrest in the G2/M phase and Tetherin/BST-2, a tetraspanin that keeps virions stuck to the cell surface without their activity being thwarted by the viral accessory protein Vpu (42, 101). The spread of HIV-1 from macrophages to T cells is facilitated by Vpr through extensive transcriptomic changes by targeting PU.1, which is a transcription factor essential for gene expression during a host innate immune response (106). A study has shown that the Vpr-Nef-Env axis considerably reduces the mannose receptor (MR), a macrophage restriction factor, to facilitate HIV infection by preventing their action (107). An *ex-vivo* study on primary human monocyte-derived macrophages (MDM) showed that Human beta-defensins (hBD2 and hBD3) inhibit HIV replication in a dose-dependent manner and hBD2 through G protein-coupled receptor (GPCR) signaling, suppresses HIV at an early stage (108). Finally, the macrophages’ means of defense are challenged by the virus via its accessory and envelope proteins. This is a form of intelligence used by the virus to prevent the effect of host restriction factors. Through the coordinated action of Vif, Vpr, Vpu, and Env, HIV systematically dismantles the restriction landscape of macrophages, converting these immune effectors into durable viral sanctuaries and rendering curative intervention dependent on strategies that can reach and reactivate latent provirus across diverse tissue compartments.

## 5 Future research direction on macrophage polarization and HIV reservoir

Research into a cure for HIV must also evolve and focus on understanding how the provirus establishes latency in macrophages, a process that, unlike its CD4+ T cell counterpart, remains incompletely characterized at the mechanistic level. Macrophage-specific biology diverges significantly from what is known in T cell reservoirs, making direct translation of existing T cell-based approaches insufficient.

Therefore, future research must adopt a myeloid-specific framework, that addresses four strategic priorities: latency modeling, immunometabolic regulation, latency reactivating agent efficacy across macrophage phenotypes, and cytotoxic dynamics (5, 109–111).

### 5.1 Latency models for macrophages

A critical obstacle to macrophage reservoir research is the scarcity of validated latency models, compared to the diverse and well-characterized models established for CD4+ T cell reservoirs. The existence of few latency models for macrophages is of particular interest for basic science research. A recent study has shown that the M4 phenotype (IL-1R+S100A8+MMP7+) is the main reservoir of HIV in the urethral mucosal stroma in patients receiving ART and has shown that LPS can reactivate the latent virus (20). A cell model with latent HIV that could serve as a tool for further research on this subtype of M4 macrophage has not yet been revealed. We know that sexual intercourse is the main route of transmission for the virus and that urethral M4 macrophages constitute a silent active reservoir at this level and can easily contribute to the virus transmission. A further complication is that macrophage provirus research requires primary monocyte isolation or THP-1 differentiation before infection with pseudoviral reporter constructs; a technically demanding workflow that itself limits the availability and throughput of latency screening. Only one type of THP-1 cell, the HIVGKO provirus model for macrophage latency, is currently available (112). Another model is U1, which is a post-integrative latency model; it exhibits a Tat function defect that can be corrected by exogenous addition of this protein (113). In summary, while the HIVGKO and U1 models provide a starting point for shock-and-kill and block-and-lock strategies, neither captures the M4 phenotype or tissue-specific reservoir dynamics now recognized as clinically significant, making the development of new, phenotype-specific latency models a research priority.

### 5.2 Immunometabolism modulates macrophage polarization in PWH

Immunometabolic reprogramming represents a critically underexplored dimension of macrophage reservoir biology, as the metabolic shifts induced by HIV infection. Particularly the glycolysis-OXPHOS imbalance, appear to directly shape polarization state and thereby influence viral latency. Very little data exists on how immunometabolism modulates macrophage polarization in PWH and influences latency, as a better understanding of this could serve as a tool for a cure. For example, during metabolic reprogramming in HIV infection, two main metabolic characteristics appear: an increase in glycolysis and a decrease in OXPHOS. This metabolic shift is accompanied by epigenetic reprogramming, including suppression of DNA methylation and increased histone acetylation, that may directly regulate the transcriptional silencing or reactivation of integrated proviral DNA. In addition, metabolic change is the key factor in the significant secretion of pro-inflammatory cytokines, such as IL-1β, TNF-α and IL-6, which promote sustained systemic inflammation (114), favorable to HIV, and also intervene in the modulation of macrophage polarization. The convergence of metabolic reprogramming, epigenetic regulation, and cytokine-driven polarization in HIV-infected macrophages suggests that immunometabolic pathways represent both a mechanistic explanation for reservoir persistence and a potential therapeutic target. Especially for LRA strategies that could exploit metabolic vulnerabilities specific to infected macrophage phenotypes.

### 5.3 Capacity of latency-reversing agents to reactivate the virus from its latent state in different types of macrophages

While latency-reversing agents have been evaluated for their capacity to reactivate HIV in macrophages, their phenotype-specific efficacy, particularly whether M1, M2, and intermediate macrophage subtypes respond differently, remains almost entirely uncharacterized. The heterogeneity of macrophages must therefore be of interest because we don’t know how macrophage types (M1 or M2) respond to LRAs that have proven effective on CD4 T cells and M0. Indirect evidence for the clinical significance of this gap comes from studies of HIV-infected individuals on ART, where the balance of macrophage phenotypes is itself altered by infection; with M1 and M2 subsets significantly reduced in favor of intermediate Mi macrophages (65). If LRA responses are phenotype-dependent, then this infection-driven shift in polarization landscape may fundamentally alter reservoir reactivation dynamics. These findings suggest that LRA efficacy in macrophage reservoirs cannot be assessed without accounting for the polarization state of the target cells, a variable that has been systematically overlooked in existing shock-and-kill research.

### 5.4 Virus cytotoxicity on macrophages polarized non or with LRAs

Despite the known resistance of macrophages to HIV-induced cytopathic effects. The mechanisms governing virion release from virus-containing compartments (VCCs) in phenotypically distinct macrophages, and whether LRA-induced reactivation triggers productive release or cell death, remain almost entirely uncharacterized. The mechanism behind this phenomenon in macrophages remains unexplained, and there is little data on how virion release occurs in M1 and M2 macrophages. Similarly, it is important to confirm whether M1 or M2 cells forming the reservoir can readily release virions after LRA has been used to reactivate the provirus. A related and equally unexplored question concerns programmed cell death pathways in macrophages following viral reactivation. The CARD8 protein, which triggers pyroptosis in response to HIV-1 protease activity, has been shown to drive CD4+ T cell depletion during HIV/SIV infection (115). However, whether this mechanism operates comparably in macrophage subtypes remains uninvestigated. Establishing whether phenotype-specific cytotoxic and virion-release dynamics differ between M1 and M2 macrophages, both with and without LRA intervention, is essential for understanding the response of macrophage reservoirs to shock-and-kill strategies. This will help determine whether these strategies can successfully eliminate infected macrophages or merely reactivate the virus without achieving reservoir clearance.

## 6 Conclusion

This review has established that macrophages constitute a biologically distinct HIV reservoir whose persistence is governed by polarization state, tissue localization, immunometabolic reprogramming, and resistance to cytopathic effects. Furthermore, viral reservoir formation in macrophages differs from that in CD4 cells, as there are two stages of latency in macrophages: pre-integration, which is less stable, and post-integration, which is more stable. Additionally, the accumulation of mature virions in VCCs within macrophages highlights their biological difference from CD4 cells, which release virions following the cycle. Each feature represents a dimension inadequately addressed by T cell-centered cure strategies. The polarization of macrophages into M1 or M2 influences this persistence and latency. The more M1-type macrophages there are, the more resistant they are to HIV, while M2-type macrophages are less resistant. This balance of permissiveness during the macrophage immune response to HIV therefore represents a favorable factor for the establishment of the reservoir. The signaling pathways governing macrophage polarization, such as the JAK/STAT, NF-κB, and mTOR axes, represent tractable therapeutic targets for redirecting macrophage phenotype toward the non-permissive M1 state, potentially reducing reservoir permissiveness without requiring direct viral elimination. Therapy directed by targeting the provirus in polarized macrophages could help to eliminate the virus in patients. Central among the unresolved questions is how immunometabolic states within polarized macrophages regulate proviral latency and permissiveness to reactivation, an understanding that will be indispensable for designing LRA strategies and immunotherapeutic interventions capable of reaching and clearing the myeloid reservoir.
